# Comment on Balsamo et al.: “Birt–Hogg–Dubé syndrome with simultaneous hyperplastic polyposis of the gastrointestinal tract: case report and review of the literature”

**DOI:** 10.1186/s12920-022-01233-9

**Published:** 2022-04-15

**Authors:** Flávia Balsamo, Pedro Augusto Soffner Cardoso, Sergio Aparecido do Amaral Junior, Therésè Rachell Theodoro, Flavia de Sousa Gehrke, Maria Aparecida da Silva Pinhal, Bianca Bianco, Jaques Waisberg

**Affiliations:** 1 Discipline of General Surgery of Urgency and Digestive System, Department of Surgery I, Faculdade de Medicina Do ABC, Avenida Lauro Gomes, 2000, Santo André, São Paulo CEP 09060-870 Brasil; 2 Discipline of Biochemistry, Department of Morfology and Physiology, Faculdade de Medicina Do ABC, Avenida Lauro Gomes, 2000, Santo André, São Paulo CEP 09060-870 Brasil; 3 Discipline of Sexual and Reproductive Health and Populationa Genetics, Department of Collective Health, Faculdade de Medicina Do ABC, Avenida Lauro Gomes, 2000, Santo André, São Paulo CEP 09060-870 Brasil

**Keywords:** Birt–Hogg–Dubé syndrome, FLCN gene, Angiomyolipoma, Gastrointestinal hyperplastic polyposis

## Abstract

In this comment, we highlight the diagnosis of Birt–Hogg–Dubé (BHD) in a 60-year-old man was made from identification and removal of normochromic papular cutaneous lesions whose histological examination indicated trichodyscomas and which are considered equivalent to fibrofolliculomas, presence of bilateral renal mass suggestive of angiomyolipomas by imaging exams. A benign/likely benign variant of *FLCN* in the intron 13 was also detected. Still, his previous pathological history presented other relevant data such as the prior removal of vocal cord angioma, total thyroidectomy, and left parotidectomy due to a cystic lesion whose histopathological examination revealed the presence of oncocytoma and lipomatosis, in addition to basal cell cutaneous carcinoma. Simultaneous gastrointestinal hyperplastic polyposis was found in this patient. The case we reported does not have the genotypic and phenotypic expressions most present in BHDS. These facts make it important for readers to know the clinical and genetic presentation facets of this unusual syndrome.

## Background

Initially, we are grateful for the considerations offered by van de Beek and colleagues regarding our article, which allow for the discussion of this important syndrome, which, due to its etiopathogenesis, has repercussions in various organs of the human body and implies a careful clinical follow-up.

The diagnosis of Birt–Hogg–Dubé syndrome (BHDS) must be based on the fulfillment of the primary criterion (at least five fibrofolliculomas of adult-onset and with histological confirmation of at least one of them; the presence of pathogenic germline variant of the *FLCN*), or two minor or secondary criteria (multiple bilateral lung cysts, with or without spontaneous primary pneumothorax; multifocal or bilateral kidney tumor or kidney cancer of mixed chromophobic and oncocytic histology with onset < 50 years of age; first-degree relative with a BHDS). Even in patients who do not meet all the clinical diagnostic criteria listed above, confirmation of the pathogenic variant of *FLCN* would be enough for BHDS to be considered [1–5]. In the case of this study, the diagnosis was made (i) from identification and removal of normochromic papular cutaneous lesions whose histological examination indicated trichodyscomas and which are considered equivalent to fibrofolliculomas, (ii) presence of bilateral renal mass suggestive of angiomyolipomas by imaging exams. A benign/likely benign variant of *FLCN* in the intron 13 was also detected. The legend of Figures 1 and 2 of the article was mistakenly described as “fibroepithelial polyp (skin fibroma)” when, in fact, it corresponds to one of several trichodiscomas (> 5) found in the patient in this report. However, the text of the article is correct when referring to Figures 1 and 2 as “anatomopathological examination of the dorsal and frontal lesions revealed trichodiscomas.” This misunderstanding may have contributed to some of the timely comments made by van de Beek and colleagues.

### Clinical manifestation and genetic findings

BHDS can also occur sporadically in individuals with no family history of the syndrome. These individual can be carrier of de novo variant in the *FLCN* gene [6]. The clinical history of the patient in question did not mention any relative with similar manifestations. Still, his previous pathological history presented other relevant data such as the prior removal of vocal cord angioma, total thyroidectomy, and left parotidectomy due to a cystic lesion whose histopathological examination revealed the presence of oncocytoma and lipomatosis, in addition to basal cell cutaneous carcinoma. Moreover, solid bilateral renal masses suggestive of angiomyolipoma were observed on angiography. Consistently, lipomas [7–9], parathyroid adenomas [9], thyroid cancer [10, 11], oncocytoma, and parotid adenoma, and basal cell and squamous cell carcinoma [2, 7, 12], as well as often bilateral kidney tumors [3, 6, 7, 9] have also been described in patients with BHDS.

Also consistent with findings in BHDS [12, 13], the cutaneous manifestations identified as trichodiscomas were presented in the case we reported as multiple normochromic papules presenting on the face (frontal region) and dorsum. Although the most frequent lesions are fibrofolliculomas, found in more than 85% of patients over the age of 25 [10], trichodiscomas present as smooth, dome-shaped papules, measuring 2 to 4 mm in diameter, and maybe single, multiple, or merge in the form of plaques [14]. As suggested by Schulz et al. [15], trichodiscomas and fibrofolliculomas are the same lesions but sectioned in different planes, which gives rise to artificial differences in histological interpretation. The follicle involved may be the same in cases labeled as fibrofolliculoma, trichodiscoma, or even acrochordon, and it may be that these skin lesions represent evolutionary stages of a single lesion.

Immunophenotypically these dermatopathies are similar and, therefore, derived from the same histiogenic precursor, which step up this possibility [16–20]. Fibrofolliculomas and trichodiscomas have overlapping histological features, and skin biopsy with puncture rather than lamina biopsy is preferred to examine the general architecture of these lesions [2, 5, 20]. To diagnose the cutaneous lesions, in this case, resection and histopathological study were performed, which identified benign lesions (trichodiscomas) and malignant lesions (basal cell carcinoma). Although most patients with BHDS will have typical cutaneous manifestations, those without typical cutaneous manifestations are also at risk of developing renal tumors and pneumothorax [1]. About 20% of patients with HBDS will not present pulmonary manifestations; therefore, the absence of symptoms or changes in chest imaging does not make the diagnosis unfeasible [12, 18, 21].

About a third of patients with BHDS have renal involvement by tumors [2, 16], often bilateral and multifocal, with slow growth and generally asymptomatic in the early stages [22, 23], usually diagnosed in males between 46 and 52 years of age. This scenario was also observed in the present case that was male, and asymptomatic kidney tumors were diagnosed by imaging at 60 years of age. In the MRI, the appearance of solid renal lesions was very suggestive of angiomyolipomas, in addition to the presence of renal cysts. This concomitant finding has also been reported in the literature [23–25].

Colorectal tumors, benign or malignant, are not considered a frequent phenotypic manifestation of BHDS. There were no still reports of hyperplastic colorectal polyps or in other locations in the gastrointestinal tract [26]. We want to clarify that we were careful to use the term “simultaneous” and not the word “associated” when describing the gastrointestinal hyperplastic polyposis found in the patient in this report, as we reiterate that there is no evidence that hyperplastic polyposis is part of BHDS. In Individuals with BHDS without a pathogenic variant of *FLCN* have already been identified intragenic deletions and duplication in the gene [14]. In the present case, no variants were found in other studied exons (4 to 14). Nahorski et al. [27] suggested that the somatic pathogenic variants of *FLCN* in patients with colorectal cancer are of the “transient” type and not pathogenic variants of the driver mutations. Thus, it is possible that the higher or lower risk of polyps or colorectal neoplasia in BHDS may be caused by different allelic variants of *FCLN* [14].

The tuberous sclerosis complex is known to be caused by dysregulation of the mTOR pathway, and due to phenotypic similarities with BHDS, the involvement of the mTOR pathway has been suggested to be implicated in the pathogenesis of the syndrome, although this is not the only signaling pathway implicated in the tumor suppressor action of *FLCN* [2, 4, 5, 28]. Some studies raise the question of why *FLCN*, a tumor suppressor gene, is a positive effector of the mTOR pathway. A possible explanation is that FLCN deficiency could suppress mTORC1 activity and may push other pathways to depletion, compensating the inhibition of the mTOR pathway [20], and which could explain the phenotypic similarity between BHDS, Cowden syndrome, tuberous sclerosis, and Peutz-Jeghers syndrome [4, 19, 29].

## Conclusion

The case we reported does not have the genotypic and phenotypic expressions most present in BHDS, as observed by other authors [30–34]. These facts make it important for readers to know the clinical and genetic presentation facets of this unusual syndrome.

## Data Availability

Not applicable.

## Abbreviations

BHDS: Birt–Hogg–Dubé syndrome
*FLCN*: Folliculin gene
MRI: Magnetic resonance imaging
mTOR: Mammalian target of rapamycin
